# The role of circulating cell-free DNA as an inflammatory mediator after stroke

**DOI:** 10.1007/s00281-023-00993-5

**Published:** 2023-05-22

**Authors:** Stefan Roth, Saskia R. Wernsdorf, Arthur Liesz

**Affiliations:** 1 Institute for Stroke and Dementia Research (ISD), University Hospital, LMU Munich, Munich, Germany; 2 Munich Cluster for Systems Neurology (SyNergy), Munich, Germany

**Keywords:** Cell-free DNA, Tissue injury, Stroke, Inflammation, Inflammasome

## Abstract

Stroke is the second leading cause of death worldwide and a leading cause of disability. Clinical and experimental studies highlighted the complex role of the immune system in the pathophysiology of stroke. Ischemic brain injury leads to the release of cell-free DNA, a damage-associated molecular pattern, which binds to pattern recognition receptors on immune cells such as toll-like receptors and cytosolic inflammasome sensors. The downstream signaling cascade then induces a rapid inflammatory response. In this review, we are highlighting the characteristics of cell-free DNA and how these can affect a local as well as a systemic response after stroke. For this purpose, we screened literature on clinical studies investigating cell-free DNA concentration and properties after brain ischemia. We report the current understanding for mechanisms of DNA uptake and sensing in the context of post-stroke inflammation. Moreover, we compare possible treatment options targeting cell-free DNA, DNA-sensing pathways, and the downstream mediators. Finally, we describe clinical implications of this inflammatory pathway for stroke patients, open questions, and potential future research directions.

## Cell-free DNA characteristics in health and disease

Cell-free DNA (cfDNA) is continuously released across all organs. cfDNA was first detected and described in blood plasma of healthy and sick individuals in 1948 [1]. cfDNA can be found in plasma [1], but also other body fluids including urine [2], cerebral spinal fluid [3], pleural fluid [4], and also sputum [5].

Previous studies suggest that most of the plasma cfDNA is originating from the hematopoietic system in healthy individuals [6]. In a number of conditions of altered tissue composition, for example, during pregnancy, organ transplantation, or in cancer, additional cfDNA can be released by affected tissues into the circulation [7]. Detection of differences in blood cfDNA has been proposed as a potential non-invasive diagnostic technique. For this, a variety of technologies have emerged in the past years to use cfDNA for non-invasive prenatal testing [8–11], organ transplantation [12, 13], immune diseases such as systemic lupus erythematosus [14, 15], tissue injuries such as stroke [16–19], and also cancer [20, 21].

**Fig. 1:**
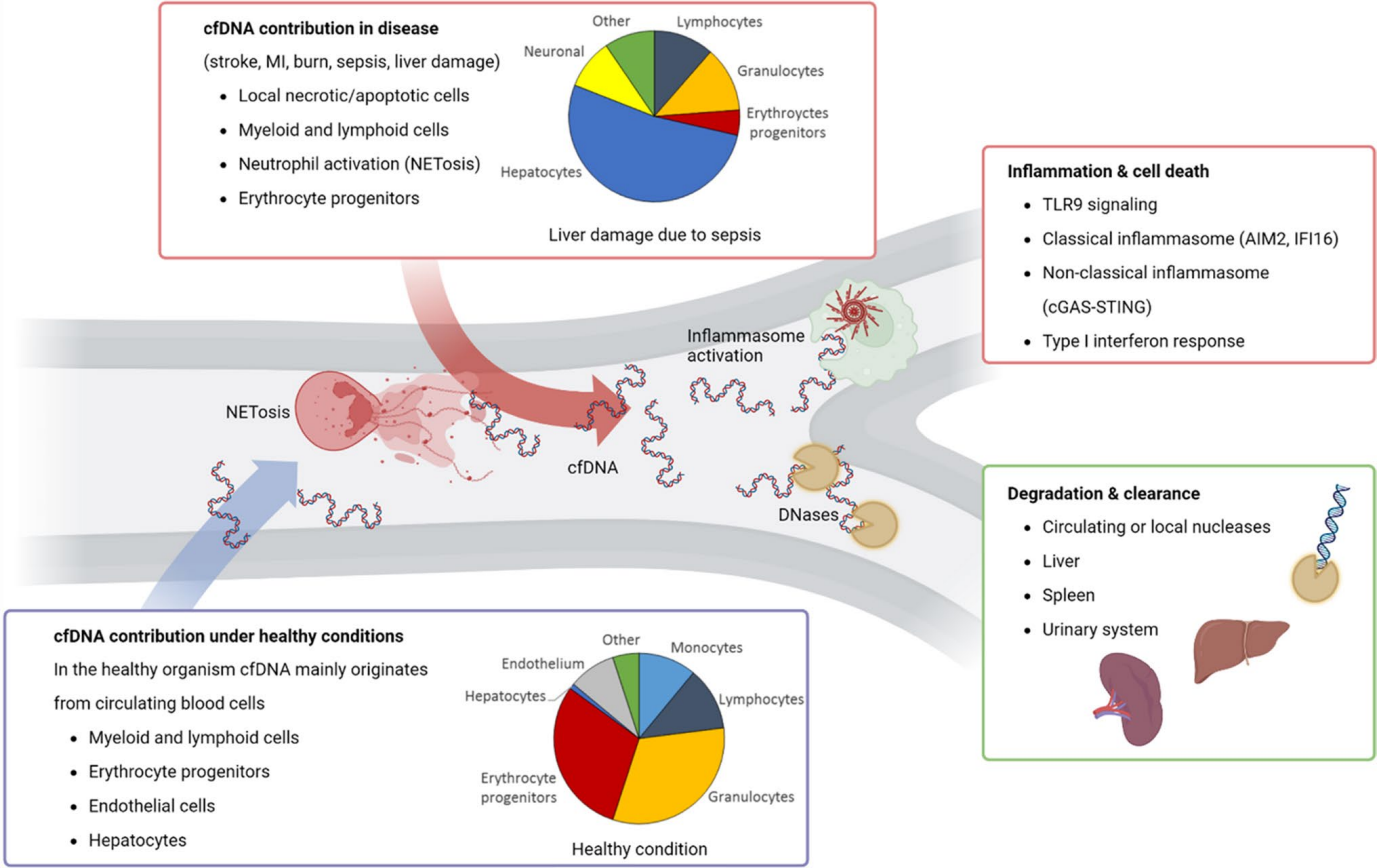
Cell-free DNA in health and disease. Under healthy conditions, cfDNA, with fragment sizes mainly ranging in multiples of 150–170 bp, is found in the blood circulation. These DNA fragments originate mainly from apoptotic erythrocyte progenitors, white blood cells, and endothelium. Degradation of cfDNA is achieved by circulating and local nucleases, e.g., DNase 1 and intracellular nucleases, or DNase 2 in the lysosome. Spleen, liver, and the urinary system facilitate the removal of cfDNA [27]. Under pathological conditions, here exemplified by septic liver damage, the origin of cfDNA depends on the organ of disease and the systemic immune response to the organ injury. For the given example of septic liver damage, cfDNA originates mainly from hepatocytes but also white blood cells [23]. Increased levels of cfDNA cannot be degraded sufficiently [28], which then leads to innate immune activation via TLR- or inflammasome-signaling [29–31]

Recent studies were able to answer another important question, the origin of cfDNA in health and disease (Fig. 1). Based on the fact that dying cells release cfDNA, each tissue provides a unique DNA methylation pattern [22]. Two methods were used to analyze the methylation pattern: first, detection of CpG nucleotides based on bisulfite conversion and subsequent arraying on a bead chip [23, 24], which enabled to cover up to 290,000 CpGs locations per sample [25]. Second, deep sequencing of cfDNA to generate a genome-wide map of the in vivo nucleosome. The data correlated with the nuclear architecture, suggesting that it could inform the cell type of origin [26]. These approaches confirmed that cfDNA originates mainly from the hematopoietic system [6] including erythrocytes, granulocytes, monocytes, and lymphocytes under healthy conditions [23, 26]. Under pathological conditions, however, affected tissue contributes to circulating cfDNA [7], such as hepatocyte-derived cfDNA was detected in the plasma samples of patients with septic liver damage [23] (Fig. 1).

Independent of the source, all cfDNA have some common properties that characterize their function and (limited) diagnostic use. All can be found non-capsulated in body fluids and are highly fragmented single- and double-stranded DNA. Plasma cfDNA consists of a mixture of different-sized DNA fragments, with fragment sizes mainly ranging in multiples of 150–170 bp—due to the nucleosome packaging, consisting of approximately 150 bp of DNA coiled around the histones [32, 33]. Based on quantitative studies, the concentration of cfDNA in healthy individuals is in average 70 ng/ml [34–40].

The concentration of blood cfDNA is mainly regulated by its degradation kinetics which can vary greatly between physiological conditions. cfDNA is rapidly degraded in body fluids; however, the reported half-life of cfDNA varies massively between conditions. In the context of hemodialysis, cfDNA half-life of 4 to 10 min was reported [41]. In the context of fetal-derived cfDNA, half-life of 1 h up to 12 h was observed [42]. Under healthy conditions, degradation and clearance are mainly performed by circulating nucleases, such as the pH-neutral DNase 1, but also active uptake by the reticuloendothelial system in liver and spleen [27] (Fig. 1).

## Cell-free DNA in stroke

Ischemic stroke is a sterile tissue injury caused by occlusion of a brain-supplying artery. The lack of oxygen and glucose leads to a necrotic cell loss and injury to tissue integrity. Inflammation is a key element of the pathobiology of stroke and the immune system actively participates in tissue damage caused by the initial ischemia [43]. Stroke does lead not only to a devastating inflammatory milieu in the compartment of the brain itself but also to a systemic inflammatory response immediately after the ischemic injury [44]. Damage-associated molecular patterns (DAMPs) are essential mediators of this systemic inflammatory response to stroke. DAMPs are a heterogeneous group of immunogenic molecules including ATP, various proteins, but also DNA and RNA [45]. DAMPs are generally secreted by stressed, damaged, or dying cells [46]. In stroke, it is supposed that DAMPs are mainly released from post-ischemic necrotic and apoptotic brain tissue [47]. The initial release of DAMPs leads to activation of the cerebral endothelium and the subsequent recruitment of local and circulating immune cells which are amplifying the local immune response [48]. Considering the plethora of different DAMP-molecules released to the circulation after stroke, exploiting their value as non-invasive biomarkers to diagnose stroke and predict its outcome is still the goal and challenge of a large body of work in the field of stroke biomarker research [49–51]. Blood biomarkers for stroke should ideally enable to distinguish not only between stroke and stroke mimics but also between different stroke subtypes and ultimately enable rapid clinical interventions. None of the so far identified biomarkers could sufficiently fulfill these criteria and thereby so far not in clinical practice.

Since blood cfDNA levels are elevated in a variety of physiological and pathological processes [27], it is surprising that blood cfDNA is a fairly specific and accurate biomarker in stroke. Several studies were able to not only improve methodology to reliably quantify cfDNA [16] but furthermore distinguish stroke from stroke mimics [52] and even ischemic from hemorrhagic stroke [17, 38] using cfDNA-based biomarker analysis. More than 10 observational trials have quantified blood cfDNA concentrations in stroke patients and correlated its blood concentration to other blood biomarkers and/or clinical outcome parameters (Table 1). In most of these clinical studies, cfDNA was considered as a potential predictive marker of chronic outcome after stroke. In 2003, Rainer and colleagues were the first, using quantitative real-time PCR for the β-globin gene, to show that blood cfDNA concentration is increased already 3 h after stroke. This quantitative analysis was sufficient for appropriate early risk stratification and prediction of 6-month disability and mortality after stroke [16]. Across all clinical studies, cfDNA quantification was performed using either quantitative real-time PCR (results provided in kilogenome-equivalent/L) or by fluorometric/spectrometric methods (results provided in arbitrary units or ng/ml). Most studies identified a general increase of cfDNA blood concentration early after stroke (Table 1). However, sampling time between studies varies largely (3–48 h after symptom onset) and is also insufficiently described. Correspondingly, reported cfDNA concentrations are differing dramatically between studies either due to these differences in sample acquisition after stroke or because of other unreported technical differences: healthy control (284.7 ng/ml ± 462.9 // 1436.9 ± 1326.9 kilogenome-equiv./L in healthy controls and 658.5 ng/ml ± 883.8 // 3025.3 ± 2589.4 kilogenome-equiv./L in stroke patients (Table 1 [16, 17, 19, 28, 37, 38, 53–55]). Geiger and colleagues [18] chose a different way and quantified blood nucleosome cfDNA (On d 3: Barthel Index ≥ 50: 523 AU vs Barthel Index < 50: 869 AU), for which blood concentrations correlated well with the function outcome of stroke patients. Yet, only few studies provide information about kinetics of cfDNA concentration after stroke. One study performed repetitive measurements throughout the first 72 h after stroke onset and observed the highest concentration of blood cfDNA at 48 h after symptom onset [38]. Geiger et al. followed up elevated cfDNA levels for a week after stroke [18]. They were able to show a correlation between nucleosome cfDNA and infarct volume 3 days after stroke. Moreover, the kinetics of nucleosome cfDNA concentration correlated well with the clinical status (Barthel Index).

**Table 1:** Stroke studies analyzing cfDNA as a potential biomarker. *Abbreviations*: ctrl = control; dsDNA = double-stranded DNA; mRS = modified Rankin score; WBC = white blood cells

Year	Author	Sample number	DNA quantified	Time of sample acquisition	Patient follow-up	cfDNA content control	cfDNA content stroke	Method	Additional immunological parameters
2003	Rainer TH	Human (88)	Blood cfDNA	3 h after symptom onset	6-month survival	mRS < 2: 1.0 ± 0.5 × 10^3^ kilogenome-equiv./L	mRS > 2: 1.3 ± 1.3 × 10^3^ kilogenome-equiv./L	Real-time quantitative PCR assay for β-globin gene	x
2006	Geiger S	Human (63)	Blood cfDNA	24 h after symptom onset	7 d after hospitalization	Barthel Index > 50: 523 AU (day 5 maximum)	BartheI Index < 50: 869 AU (day 3 maximum)	Nucleosome: cell death detection ELISA	S100 protein level
2006	Lam NYL	Human (44)	Blood cfDNA	24 h after symptom onset	6-month survival	~ 1(0.5–2) × 10^3^ kilogenome-equiv./L	PMRS 0–2: ~ 1 (0.35–4.5) PMRS 3–6: ~ 1.5 (0.8–9.0) × 10^3^ kilogenome-equiv./L	Real-time quantitative PCR assay for β-globin gene	S100 protein level
2007	Geiger S	Human (63)	Blood nucleosome	24 h after symptom onset	7 d after hospitalization12-month survival	~ 10–100 ng/ml nucleosomes	~ 80–800 ng/ml nucleosomes	Cell-Death-Detection ELISAPlus	Neuron-Spec. Enolase, S100 protein, CRP
2007	Rainer TH	Human (197)	Blood cfDNA	24 h after symptom onset	6 month survival	No stroke: 1050 kilogenome-equiv./L	Hemorrhagic: 1725 ischemic: mRS > 2 vs. mRS ≤ 2 1350 vs 1025 kilogenome equiv./L	Real-time quantitative PCR assay for β-globin gene	S100 protein level
2011	Tsai N	Human (100)	Blood cfDNA	48 h after symptom onset	30-d follow-up	NucDNA: 3681 ± 197 MitDNA: 1949 ± 167 kilogenome-equiv./L	NucDNA: 5393 ± 454 MitDNA: 3045 ± 384 kilogenome-equiv./L	Real-time quantitative PCR assay for β-globin gene	WBC and Platelet count
2016	Bustamante A	Human (69)	Blood cfDNA	4.5 h after symptom onset (before tPA)	48-h improvement	153.5 (66.9–700.5) kilogenome-equiv./L	408.5 (179–700.5) kilogenome-equiv./L	Real-time quantitative PCR assay for β-globin gene	x
2017	O'Connell, GC	Human (63)	Blood cfDNA	4.5 h after symptom onset	x	Three-fold higher levels of cfDNA in AIS patients than in patients diagnosed as stroke mimics		TERT qPCR with GFP605 spike-in control	Neutrophil count
2017	Valles J	Human (243)	Blood cfDNA	24 h after symptom onset	12-month follow-up	324.2 ± 13.4 ng/ml	428.7 ± 40.5 ng/ml	SytoxGreen Fluorometric	citH3 values and nucleosomes (NET marker); WBC count
2018	Vajpeyee A	Human (26)	Blood cfDNA	6 h after symptome onset	3-month outcome	x	8784 (902–33,138) kilogenome-equiv./L	Real-time quantitative PCR assay for β-globin gene	x
2020	Vajpeyee A	Human (54)	Blood cfDNA	12 h after symptom onset	3-month outcome	x	8790 (729–41,170) kilogenome-equiv./L	Real-time quantitative PCR assay for β-globin gene	x
2020	Vasilyeva I	Human (68)	Blood cfDNA	admission at hospital	3, 6, 12, 24, 48, 72 h	ctrl: 20.16 ± 7.7 ng/ml	3 h: 40.2 ± 7.8 ng/ml 24 h: 50.2 ± 5.1 ng/ml 48 h: 54.7 ± 10.1 ng/ml	Nanodrop spectrophotometer	x
2020	Cui X	Human (68)	Blood cfDNA	8 h after symptom onset	x	HC: 100—300 ng/ml (75–250 bp fragment proportion up)	AIS: 200–1200 ng/ul (300–400 bp fragment proportion up)	(paired-end 100 bp) BGISEQ-500 sequencer	x
2020	Kim H	Human (155)	Blood cfDNA	retrospec. OASIS cancer study	x	Cancer control: 37.2 ± 5.0 normal control: 38.6 ± 6.6 (ng/ml)	Cancer stroke: 56.6 ± 16.7 Control stroke: 43.2 ± 7.9 (ng/ml)	Quant-iTPicoGreen dsDNA assay; Cell Death Detection-ELISA Kit	x
2022	Grosse G	Human (92)	Blood cfDNA	24 h after symptom onset	7-d secondary timepoint	Sufficient reperfusion: 0.209 ± 0.094 µg/ml Baseline favorable outcome: 0.2 µg/ml 7-d favorable outcome: 0.32 µg/ml	Insufficient perfusion: 0.226 ± 0.124 µg/ml baseline unfavorable outcome: 0.24 µg/ml 7 d unfav. outcome: 0.48 µg/ml	Quant-iTPicoGreen dsDNA assay	Cytokines multiplex, baseline and 7d, WBC count

Tsai and colleagues established the isolation of nucleus- versus mitochondrial-derived cfDNA, since it was reported that mitochondrial cfDNA can activate different inflammatory pathways than nuclear cfDNA [56]. However, both concentrations were elevated 48 h after stroke and the proportion between nuclear and mitochondrial DNA did not change [55]. Bustamante et al. [54] used cfDNA to predict short-term neurological outcome after treating stroke patients with tissue plasminogen activator (tPA). They were able to show that patients admitted to hospital within 4.5 h after stroke onset had increased cfDNA blood concentrations compared to healthy controls. Moreover, they observed a trend of lower cfDNA levels in patients who had improved neurological status after tPA therapy. Grosse and colleagues [28] were able to show that higher levels of plasma cfDNA were associated with worse 90-day outcome and increased mortality after revascularization in stroke patients with endovascular therapy [57, 58].

Several studies aimed at correlating blood cfDNA concentrations with other blood biomarkers. Geiger and colleagues [19] performed a comparison of blood nucleosome cfDNA and other potential stroke biomarkers such as neuron-specific enolase (NSE), S100 protein, and C-reactive protein in ischemic stroke patients. Correlations were found between stroke severity at hospital admission with blood concentrations of cfDNA, NSE, and S100 at 3 days and 6 days after stroke. The same blood biomarkers were also correlated with infarct volume and the long-term recovery index after 12 months. In contrast, CRP concentrations—a widely used biomarker for systemic inflammation in numerous studies—was only correlated with stroke severity at admission but had no predictive value. Although S100 protein correlated well with a number of clinical parameters, only nucleosome cfDNA retained its prognostic value with 100% specificity for the 12-month outcome [19]. Also, Tsai and colleagues compared the predictive value of cfDNA and S100 blood concentrations [55]. Blood cfDNA levels were increased in patients with severe deficits (mRS 3–6) compared to mild or no deficits (mRS < 2). Although blood was only sampled once during admission to the hospital, blood cfDNA concentration was a predictor of stroke outcome. In contrast, S100 protein concentrations in this study was not correlated with stroke outcome.

Taken together, cumulative evidence from a considerable amount of studies by now suggests that quantification of blood cfDNA concentrations might be at least equivalent in its diagnostic value in comparison to much more commonly investigated protein biomarkers such as CRP and S100 proteins. Specifically, current data indicates that the analysis of cfDNA concentrations might be particularly suitable to predict long-term clinical outcome for which blood cfDNA concentrations showed a good predictive value while other biomarkers correlated mainly to short-term disease severity or clinical endpoints. While the quantitative real-time PCR for β-Globin [16] and TERT [52] provides high sensitivity in detection of cfDNA fragments, meanwhile, more common use of (fluoro)spectrometric methods has practical advantages considering the speed and broad availability of these methods—potentially also as point-of-care devices to be used for acute stroke patients to aid rapid diagnosis and decision making. Despite this promising results on the value of cfDNA as a clinical biomarker, future trials are warranted to address key open questions and technical caveats. These include the development of more sensitive and standardized cfDNA analysis methods, the assessment of its use across plasma and serum samples, and, importantly, the description of the cfDNA blood concentration kinetics in stroke patients after onset of tissue ischemia.

## Immune sensing of cfDNA

### DNA uptake

**Fig. 2:**
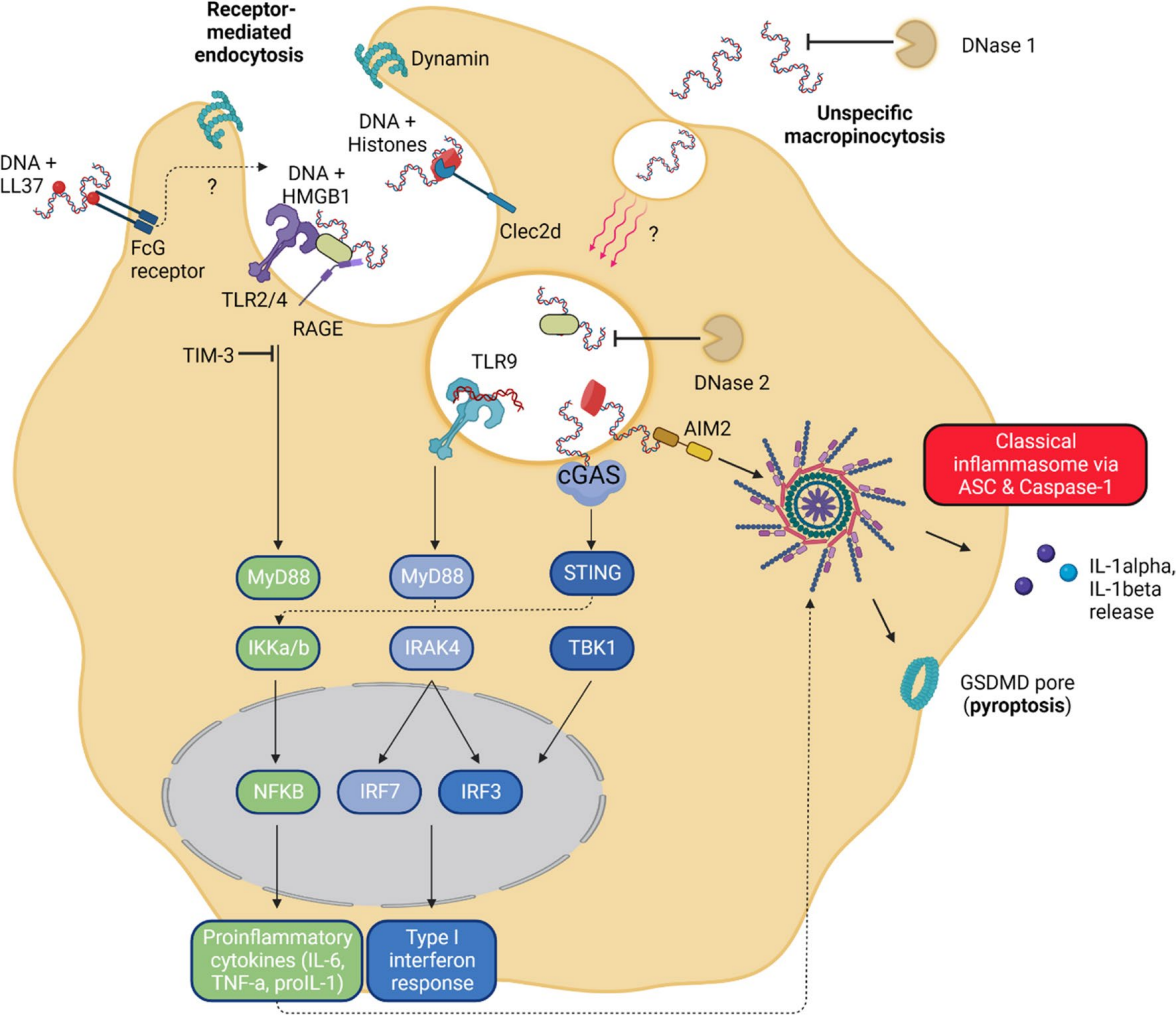
Immune sensing of cfDNA. On the cell surface, pattern-recognition receptors, such as TLRs and RAGE, can sense DNA-bound proteins and peptides. It is known that HMGB1, histones, but also the antimicrobial peptide LL37 can help mediating active DNA uptake via receptor-mediated endocytosis. TLRs and RAGE do not only mediate the uptake of DNA but also activate a proinflammatory cascade via IKKα/β or IRAK4 leading to NF-κB or IRF7 translocation to the nucleus. A minority of naked DNA can also be uptaken by unspecific micropinocytosis. Endosomal cfDNA will be sensed by TLR9 or degraded via nucleases from the lysosome and is then sensed by the cytosolic DNA sensors cGAS and AIM2. cGAS drives mainly an interferon Type I response via TBK-1 phosphorylation. AIM2 leads to classical inflammasome assembly. ASC is recruited and cleaved caspase-1 in the end leads to secretion of active IL-1β and pore formation via Gasdermin D

The immunogenic properties of nucleic acids are known for nearly 60 years [59, 60]. Translocation of DNA to the cytosol represents a potent trigger for the immune system, driving production, and secretion of proinflammatory cytokines such as IL-1β, IL-18, and interferons. DAMP-induced immune responses include expression of Type I and II interferons, transcription of pro-inflammatory genes, activation of inflammasome cascades, but also the induction of autophagy and the initiation of cell death pathways (Fig. 2) [29].

Entry of cfDNA into the cytosol is dependent on its characteristics. DNA modifications, such as methylation (CpG) and DNA-bound proteins, increase the affinity to pattern recognition receptors (PRRs) [61]. For example, DNA derived from activated neutrophils (NETosis) or necrotic cells provide DNA-bound proteins. Characteristic NET proteins are neutrophil elastase (NE), myeloperoxidase (MPO), histones, and the antimicrobial peptide LL37/CRAMP [62]. Also, cell death–derived cfDNA is bound to characteristic proteins including histones and “HMGB1 [63]. Physiologically, these proteins provide nuclear functions such as DNA bending and packaging in the nucleus [64].

Lu et al. [65] and others observed that naked cfDNA from activated lymphocytes in systemic lupus erythematosus (SLE) binds only with low affinity and induces unspecific macropinocytosis. However, binding of cfDNA to extracellular HMGB1 leads to a specific clathrin-/caveolin-1-dependent receptor-mediated endocytosis of the HMGB1-cfDNA complex. The enrichment of DNA with histones and/or HMGB1 leads to binding to cell membrane-located PRRs [61, 66]. The DNA uptake pathway is induced by the binding of HMGB1 to TLR2 and TLR4, ultimately leading to a proinflammatory response of the cells releasing TNF-α and IL-8 [67]. Another receptor known to bind HMGB1 is the receptor for advanced glycation endproducts (RAGE). It was shown that RAGE mediates HMGB1 dynamin-dependent endocytosis. Moreover, DNA-bound histones can bind to Clec2d, a membrane-bound C-type lectin receptor. The binding of the K-rich histone tail to Clec2d leads to endosomal uptake of the histone and its nucleic acids. Furthermore, endolysosomal degradation then enables DNA recognition via TLR9, which is located in the endolysosome, and other DNA sensors [68]. Moreover, LL37, a peptide bound to NET-DNA [62], not only provides antimicrobial properties but also increases the affinity of DNA to membrane-bound receptors [69]. LL37 is described to increase the immunogenicity of extracellular DNA into a potent ligand driving a TLR9-dependent IFN response [70]. LL37 can bind cfDNA from necrotic cells forming aggregates and condensed structures. This condensation protects DNA from nuclease degradation and can improve the uptake of cfDNA by macrophages and dendritic cells [71]. Also, inhibitory receptors affect uptake and sensing of cfDNA. T cell immunoglobulin and mucin domain containing (TIM)-3 limit the activation of Cyclic GMP-AMP synthase (cGAS), and thereby signaling via the stimulator for interferon genes (STING) pathway by suppressing endocytosis of extracellular DNA. Blockage of TIM-3 led to higher efficacy in cancer immunotherapy trials via increased interferon and suppressed tumor growth factor expression [72].

In summary, naked cfDNA only binds membrane-bound PRRs with low affinity and induces unspecific uptake [65]. cfDNA shows high potential to enter (immune) cells, when bound to nuclear proteins such as histones and HMGB1 [65–67]. Moreover, the modifications of nuclear DNA with additional proteins, as seen during NETosis in neutrophils, increased its uptake [68]. However, very little is known about the detailed mechanisms of DNA uptake and intracellular processing. Specifically, these mechanisms are virtually unknown for cfDNA release after stroke. Finally, defining the tissue-of-origin of cfDNA and thereby characterizing and understanding its tissue-specific modifications will improve the understanding of DNA uptake and its immunomodulatory functions after stroke.

### TLR9 DNA recognition

Out of the 10 TLRs being expressed in human cells, DNA can be sensed via TLR9 in the endolysosomal compartment. TLR9 sensing of DNA was the first nucleic acid sensor being identified [73]. TLR9 is a specific sensor for CpG motifs in DNA which is manifold more abundant in bacterial (and viral) DNA in comparison to mammalian DNA (Fig. 2). Binding of bacterial DNA is species-, sequence-, and DNA structure-dependent [74]. Consequently, DNA bending proteins including HMGB1 but also histones modify the TLR9 binding affinity [75]. TLR9 signaling is dependent on the DNA concentration and shows a dose–response function [76]. It preferably binds to the phosphodiester backbone of DNA, inducing receptor dimerization [77]. During cell homeostasis, TLR9 is mainly located in the endoplasmatic reticulum, and for its proinflammatory signaling, endosome shuttling is required. Similar to intracellular movement of CpG DNA, TLR9 transfers to the nucleic acid-containing structures, such as endosomes, lysosomes, and endolysosomes [78]. The dimerization of TLR9 induces a MyD88-dependent signaling cascade, leading to Nf-κB-mediated transcription of proinflammatory factors and cytokines [79]. Alternatively, TLR9 signaling can activate the transcription factor interferon regulatory factor 7 (IRF7), leading to IFN-α expression [80].

The functional role of TLR9 in ischemic injury is controversial, with some studies in experimental stroke, and myocardial ischemia models have suggested a protective function of TLR9 through the PI3K/Akt signaling pathway [81, 82]. Yet, another study in experimental myocardial infarction has shown p38 MAPK activation in response to TLR9 aggravated myocardial ischemia-perfusion injury [83]. Similarly, a report using inhibitory oligonucleotides to block TLR9 activation observed reduced ischemic brain damage with this therapeutical approach [84]. Nevertheless, blood concentrations of the DNA bending protein HMGB1 have been shown to increase after stroke and showed proinflammatory functions such as proinflammatory cytokine release [44, 85, 86]. Although it was previously described to enhance binding of TLR9 to DNA [87], HMGB1 might also have inflammation-resolving properties. It was shown that cytosolic HMGB1 binding TLR9 improves tissue repair, increased wound healing, and angiogenesis [88]. Raucci and colleagues [89] showed that protective or harmful effects of HMGB1 and TLR9 might finally dependent on the concentration of HMGB1. Hence, future studies are required to understand the detailed interaction of DNA-binding proteins to cfDNA and their impact on the resulting immunological function after tissue injury.

### AIM2 DNA sensing

The inflammasome is a high molecular weight protein complex initiating cleavage of the effector enzyme caspase-1 for an instant proinflammatory response [90]. Active caspase-1 initiates the release of the proinflammatory cytokine IL-β and membrane pore forming Gasdermin D via cleavage into its active form [91]. AIM2 is a cytosolic DNA sensor which is part of the pyrin and HIN domain protein family. AIM2 activates the inflammasome pathway in response to exogenous or endogenous cfDNA (Fig. 2) [92]. It shows preference for dsDNA over single-stranded DNA [93]. The interaction is mainly electrostatic via lysine and arginine residues matching with phosphate and deoxyribose of the DNA backbone. Interestingly, the isolated AIM2 HIN domain was found to already bind 20-bp dsDNA efficiently. However, full activation of the inflammasome via AIM2 binding required dsDNA fragment of > 80 bp sizes [94]. After the contact and binding of DNA, AIM2 recruits the Apoptosis-associated speck-like protein containing CARD (ASC), an adaptor protein forming filaments as a binding and cleavage platform for caspase-1 [95]. Subsequent recruitment of caspase-1 to this structure activates initial cleavage followed by self-cleavage of caspase-1 into active state [96]. Cleavage of caspase-1 then leads to release of cleaved proinflammatory cytokines IL-1α and IL-1β.

AIM2, together with NLRC4, contribute to inflammation and subsequent inflammatory injury after brain ischemia. Whereas NLRP3 and NOD2 deficiency did not improve outcome after experimental stroke, ASC-KO, AIM2-KO and NLRC4-KO mice had reduced infarct volumes [97]. Another study analyzed the contribution of histone deacetylase 3 (HDAC3) on inflammasome activation [98]. HDAC3 expression was increased in microglia of mice 3d after experimental stroke. This matches also with the expression peak of AIM2. In this context, it was shown that the inhibition of HDAC3 in mice not only improved the outcome, but further inhibited AIM2 inflammasome activation. The authors hypothesized that this effect was mediated due to modulation of the STAT1 pathway subsequently decreasing AIM2 expression [98]. Kim et al. found that aged mice, 6 months of age and older, show significantly increased AIM2 expression in the brain and AIM2 deficiency led to improved cognitive function. Here, AIM2 was mainly expressed in Iba-1 + microglia, but also in the endothelium and neurons with a peak of AIM2 expression 3 days after experimental stroke [40]. A post-stroke increase in AIM2 expression was also found after experimental stroke in rats [99]. Interestingly, AIM2-dependent inflammation was ameliorated by administration of the neuroprotective steroids 17β-estradiol and progesterone. Although no further mechanism for this effect is provided, the positive outcome with both gonadal hormones might be a possible anti-inflammatory treatment after brain ischemia [99]. AIM2 deficiency was shown to improve not only infarct volume and functional outcome but also the integrity of the blood–brain-barrier. Xu and colleagues [100] found more tight junction proteins, such as ZO-1 and occludin, and less endothelial adhesion molecules in the absence of AIM2. Recent results have demonstrated that AIM2 is also crucial in mediating the systemic inflammatory response to stroke. We were able to show that the increased levels of cfDNA after stroke correlate with a pronounced phenotype of immunosuppression. Binding of post-stroke double-strand cfDNA to AIM2 leads to increased blood IL-1β concentrations, which results in apoptosis of circulating lymphocytes [30].

Taken together, AIM2 plays a pivotal role in the local neuroinflammatory as well as the systemic immune response to stroke. Expression of AIM2 in neural cells, microglia, neurons, but also endothelium was increased in response to brain ischemia [40, 100]. Interestingly, similar mechanisms are observed also in aging and other brain disorders such as neurodegeneration, suggesting that DNA-mediated AIM2 inflammasome activation might represent a common therapeutic target to prevent brain pathology of multiple causes.

### cGAS DNA sensing

cGAS is another cytosolic DNA sensor with distinct functional properties leading to a rapid interferon response [101]. DNA binding of cGAS leads to the enzymatic activation of the protein producing cyclic dinucleotide 2′3′-cGAMP [102]. This cyclic dinucleotide is the ligand for the adaptor protein stimulator of interferon genes (STING), which itself can recruit TANK-binding kinase I (TBK-1) [101]. Contact of TBK-1 to STING leads to phosphorylation of serin-366 enabling binding and mobilization of the transcription factor IRF3. This interaction then eventually leads to expression of Type I interferons (Fig. 2) [29, 103]. In addition to the IRF-interferon pathway, cGAS-STING interaction does also results in NF-κB activation [104, 105]. As for DNA sensing by AIM2, also cGAS sensing of DNA is dependent on DNA length. Similar to AIM2, it was previously reported that under conditions with low DNA concentrations, long dsDNA species (> 1000 bp) are more stimulatory and have a much lower concentration threshold for cGAS activation [106]. This argues for long DNA species being the physiological trigger of DNA-induced immune responses [29]. A number of studies showed that blocking cytosolic DNA sensing via cGAS ameliorates neuroinflammation and experimental stroke outcome. Both the inhibition of cGAS by using synthetic oligonucleotides (A151) and blocking the downstream mediator STING showed similar effects of reduced inflammation [87, 107, 108]. Liao and colleagues [109] were able to show that the cytosolic cGAS pathway was upregulated in microglia after experimental stroke. Histone deacetylase 3 inhibition has been shown to ameliorate brain injury by reducing cGAS expression [109]. Another study showed that tPA-induced release of NETs by brain-infiltrating neutrophils activates the cGAS-STING pathway. This effect leads to increased release of IFN-β and IL-6 disrupting the blood–brain-barrier. Interestingly, cGAS-STING activation was inhibited by blocking the NET release (PAD4 inhibition) or degradation of DNA (via DNase 1) [110]. Finally, Shi and colleagues used CXCR4-coated versatile nanoparticles carrying A151, a cGAS inhibitor, modulating inflammation after stroke [111]. In summary, not only classical inflammasome activation via AIM2 drives neuroinflammation. The sensing of cfDNA originating from necrotic tissue and also release of NET-DNA from activated neutrophils can initiate a cGAS-STING-mediated interferon response.

## cfDNA degradation and inhibition of cfDNA sensing

### Recombinant DNase 1

A potential treatment option to prevent cfDNA-induced immunity is the therapeutic use of DNase. The DNase family divides into DNase 1 and DNase 2 with each of them containing further subtypes which can be separated by their different biological and biochemical characteristics [112]. Generally, DNase 1 is thought to be the “neutral” DNase degrading cell-free DNA in the circulation. DNase 2 is found in the lysosomes of cells'; the milieu in the lysosome implicates it as an “acidophilic” DNase [113].

In the following section, we will focus on DNase 1 as this is a compound with potential therapeutic drug use. DNase 1 is an endonuclease hydrolysing dsDNA and is mainly produced in the pancreas and salivary glands with a concentration of approximately 3 ng/ml in healthy human plasma and forms a single polypeptide chain of 260 amino acids [112, 114–117]. The actin-binding resistant rhDNase 1 variant shows increased ability to degrade DNA contrary to native DNase 1 [118]. The advantage of this DNase 1 variant is its DNA-hydrolytic activity which is similar or increased to native human DNase 1, but unlike the native DNase, it shows very low affinity to activity-inhibiting actin [118]. The inhalative formulation of rhDNase 1 is used for cystic fibrosis patients to improve lung function by cleaving extracellular DNA [112, 119, 120]. Concentrations of at least 50–100 ng/ml rhDNase in the serum are necessary to degrade cfDNA [121]. The half-life time in blood is about 3 to 4 h after intravenous application [115, 119].

In preclinical studies of burns or ischemic stroke, recombinant DNase 1 reduced inflammasome activation in splenic monocytes, leading to reduced IL-1β release [30]. This resulted in improved immunocompetence with reduced incidence of post-stroke infections [30]. Correspondingly, endogenous deoxyribonuclease activity has been shown to be inversely correlation with the cfDNA concentration after stroke [28]. Therapeutic administration of DNase 1 consistently resulted in significant reduction of blood IL-6 concentrations within 24 h after experimental stroke [122, 123].

In addition to its anti-inflammatory effect, DNase 1 ex vivo facilitates thrombolysis of platelet-rich thrombi. Interestingly, tPA-resistant thrombi with a DNA-scaffold of NETs could only be degraded by combined treatment with DNase 1 and tPA in vitro [124]. A previous report studying the impact of DNase 1 treatment on experimental myocardial infarction outcome described reduced inflammation and smaller infarcts in DNase 1 treated animals, resulting in improved left ventricular remodeling and endothelial function [125, 126]. Additionally, in preclinical deep-vein thrombosis (DVT)-models, cfDNA concentrations increased already after 6 h and DVT occurrence could be ameliorated by rhDNase 1 treatment [127].

**Fig. 3:**
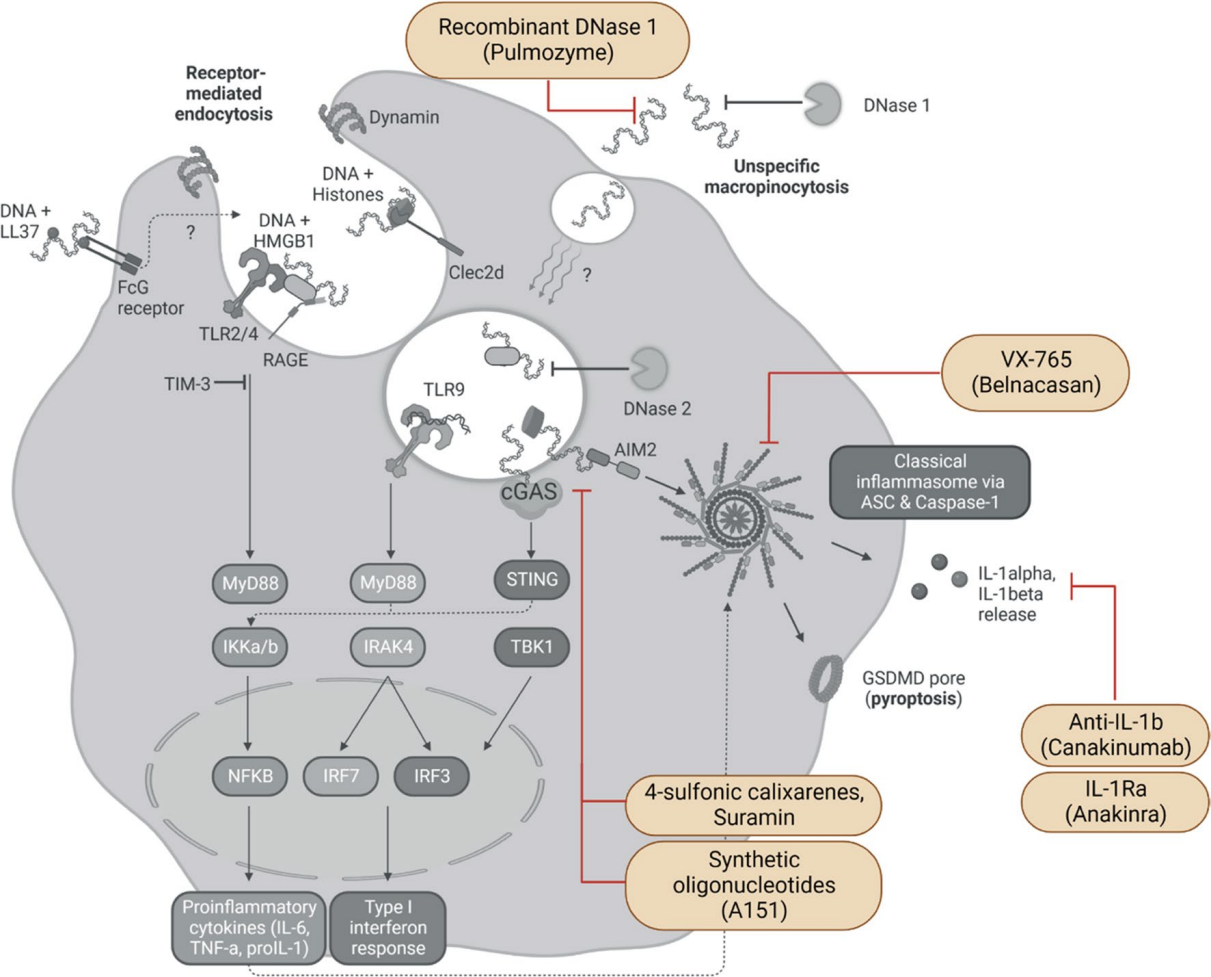
Possible treatment approaches against immunogenic cfDNA. The cellular signaling cascade of cfDNA provides a number of checkpoints for possible treatment approaches. Most upstream, cfDNA can be degraded rapidly after release to circulation by using a recombinant (human) DNase 1 [128]. Sensing of cfDNA by AIM2 can be reversibly inhibited with either 4-sulfonic calixarenes or Suramin via blocking the dsDNA binding site. Higher doses of 4-sulfonic calixarenes successfully inhibit cGAS and TLR9 [130]. Other DNA binding site inhibitors are synthetic oligonucleotides such as A151 caring a hexanucleotide motif suppressing DNA sensor signaling [131]. Another possibility is inhibition of caspase-1 with VX-765. VX-765 blocks the active cleavage site of caspase-1 impeding cleavage and secretion of IL-1. Downstream of the DNA-sensing cascade is the release of proinflammatory cytokines such as IL-1β [30]. IL-1β can be efficiently neutralized with specific monoclonal antibodies [132] or a competitive antagonist (IL-1Ra) [133], both preventing binding to IL-1 receptors

Taken together, treatment with rhDNase 1 showed two potential advantages compared to the recent post-stroke therapy. First, degrading circulating cfDNA reduces the systemic inflammatory response to stroke (Fig. 3). DNase treatment improved susceptibility against infections as well as inflammation-induced recurrent ischemic events in an atherosclerotic mouse model [31, 128]. Second, combined treatment with DNase 1 in addition to thrombolysis can improve lysis efficacy and enables to dissolve DNA-rich, tPA-resistant thrombi [127, 129]. Clinical trials are in preparation or already recruiting for both therapeutic concepts. Currently, two clinical studies (ClinicalTrials.gov Identifier NCT05203224 and NCT04785066) investigate the intravenous effect of rhDNase 1 regarding the recanalization after thrombectomy and reperfusion after ischemic stroke.

### 4-Sulfonic calixarenes

We have previously demonstrated that AIM2 inflammasome activation by cfDNA release after stroke and burn injury leads to IL-1β release [30]. This signaling pathway can be blocked by the competitive AIM2 inhibitor 4-sulfonic calixarenes [130]. 4-sulfonic calixarenes dose-dependently inhibited AIM2-dependent cell death and IL-1 β release but also the cGAS-dependent Type I interferon response and TLR9 signaling were abrogated [130]. Consequently, treatment with 4-sulfonic calixarenes attenuates post-stroke immune alterations including AIM2-dependent post-stroke immunosuppression [130]. In addition, 4-sulfonic calixarenes potently reduced post-stroke atheroprogression and recurrent ischemic events in a preclinical study (Fig. 3) [31]. Similar effects were found by treating with Suramin — a compound used to treat trypanosomias infections — a dose-dependent, reversible inhibitor of DNA sensors [130].

### Suppressive oligodeoxynucleotides

Certain DNA sequences such as the TTAGGG repeat motif, commonly found in mammalian telomeric DNA, can efficiently suppress innate immune activation [131]. Synthetic, suppressive oligodeoxynucleotides (ODN), such as A151 (a ssDNA species with four repeats of the TTAGGG motif), are competitively binding to AIM2 and other cytosolic DNA sensors (Fig. 3). The inhibition was interrupting inflammasome assembly and downstream mediator release [131]. Preclinically, ODNs showed great potential in suppressing inflammation [131]. A151 did not interfere with NLPR3 and specifically inhibited the sensing of cytosolic DNA by AIM2 [131]. A151 treatment resulted in reduced expression of cGAS, AIM2, IL-1β and IL-18 after experimental stroke and decreased infarct volume and improved neurological deficits after stroke [87].

## Downstream inhibition of DNA sensing-pathways

### Anti-IL-1β antibody *(Canakinumab)* and IL-1R antagonist *(Anakinra)*

The interleukin-1 family, including 11 cytokines, is primarily associated with innate immunity and part of the early immune response [134]. Blood concentrations for IL-1α and IL-1β, two prototypical members of the IL-1 family and early-released cytokines, are increased after stroke [133]. They are binding to IL-1 type I and II receptor (IL-1R1 and IL-1R2) activating a proinflammatory downstream signaling cascade leading to translocation of the transcription factors NFκB and AP-1 [135]. In previous studies, high systemic levels of the proinflammatory cytokine IL-1β have been associated with cytokine-induced sickness behavior after experimental stroke (Fig. 3) [136].

A clinical trial (CANTOS) for IL-1β neutralization analyzed the efficacy of this approach to prevent recurrent cardiovascular events [132]. Patients with myocardial infarction and a CRP of ≥ 2 mg/l receiving IL-1β-specific neutralizing antibodies presented significantly lower rates of recurrent cardiovascular events and non-fatal stroke [132]. However, IL-1β neutralization also led to an increased incidence of fatal infections [132], raising the need for more specific interventions without resulting in a potentially life-threatening immunosuppressive state.

The endogenous IL-1 receptor antagonist (IL-1Ra) acts as a competitive inhibitor of IL-1α and IL-1β by specifically binding the IL-1 receptor without causing biological effects [133]. Although there had been only a limited range of IL-1Ra studies [137], recombinant IL-1Ra in animal stroke models did show positive effects on ischemic lesion size and improving neurological outcome [133, 138, 139]. One Phase 2 trial for intravenous IL-1Ra administration to acute stroke patients found a reduction in inflammatory markers including neutrophil and total leukocyte counts, IL-6 blood concentration, and also led to improved clinical outcome [140]. Interestingly, subcutaneous IL-1Ra application in ischemic stroke patients within 5 h of symptom onset in the SCIL-STROKE Phase 2 trial also observed a reduction of IL-6 and CRP after treatment without reporting any safety concerns [141]. Together, both Phase 2 trials randomized only a small number of patients but suggest a potentially beneficial and safe use of IL-1Ra for stroke patients [140, 141].

## Clinical implications for stroke patients

### Infections through immunosuppression

Major tissue injury, such as stroke, induces a period of immunosuppression similar to sepsis-induced immunosuppression [30, 142, 143]. Post-injury immunosuppression is characterized by a rapid loss of T cells due to apoptosis [144]. This phenomenon predisposes the patients with local injury to systemic infections [145]. Already during hospitalization, up to 22.7% of patients with ischemic stroke present infections, especially urinary tract infections (11.5–24%) and pneumonia (10–22%) [146–149]. Finally, these infections increase mortality in stroke patients [43]. Current clinical strategies to combat stroke-associated infections are based on treatment with broad-spectrum antibiotics once the infection has already developed and been clinically diagnosed. A clinical trial (STROKE-INF) tested the paradigm of prophylactic antibiosis in stroke patients with dysphagia; however, no improvement in pneumonia outcome after stroke was found [150].

Herein, understanding the systemic immunological processes after ischemic stroke can provide the chance to switch therapy against infections from antibiosis to preventively modulating the systemic immunosuppression. We have previously shown that the release of cfDNA from dying tissue or activated neutrophils leads to a rapid systemic AIM2-mediated inflammasome activation and subsequent increased levels of circulating IL-1β after stroke. IL-1β drives the induction of substantial T cell death by stimulating macrophages to express cell-death receptor ligands (FasL/CD95L) and induce extrinsic apoptosis [30]. This newly identified pathway includes a multitude of druggable checkpoints, such as blockage of IL-1β or the inhibition of caspase-1. However, degradation of cfDNA, and thereby an upstream inhibition of the initiation of the cfDNA-driven immunological cascade, might be a promising treatment approach to restore the immune competence after tissue injury.

### Atherosclerosis

Large-artery atherosclerosis (LAA) is one of the main causes for ischemic stroke. Moreover, risk of recurrent vascular events after LAA-caused stroke is high [151]. A systemic review and meta-analysis reported a pooled recurrent stroke risk of 11.1% at 1 year [152, 153]. Especially in the early phase after stroke, recurrence rates were markedly higher in patients with LAA compared to other stroke etiologies [31]. It was shown before that the systemic inflammatory response after stroke further exacerbates atheroprogression [128]. Atherosclerosis itself is a chronic inflammatory disease, where immune cells significantly contribute to progression and vulnerability [154]. Recent studies showed that amelioration of the inflammatory milieu impairs atheroprogression. Anti-IL-1β treatment and NLRP3 inflammasome inhibition in atherosclerotic mice reduced the invasion of leukocytes to atherosclerotic plaques, resulting in reduced overall size of the plaque and reduced vulnerability to rupture [155]. Additionally, the deficiency or inhibition of cfDNA-sensing by AIM2 improved atherosclerotic plaque stability by reduction of IL-1β expression [156]. Consequently, the AIM2 inflammasome is a likely candidate for further development as a target for precision medicine in atherosclerosis [157]. We have recently demonstrated that the release of cfDNA after ischemic stroke is a potent driver of rapidly evolving plaque vulnerability and subsequent recurrent events [31]. In a newly developed model of rupture-prone carotid-plaques in combination with contralateral experimental stroke, we were able to show that the initial trigger for recurrent vascular events is cfDNA. Degradation of cfDNA by in vivo administration of DNase 1 significantly decreased plaque growth and prevented atherosclerotic plaque rupture leading to recurrent ischemic events [31].

## Summary

In the last decade, the role of the immune system gained significant importance as a key player in pathophysiological changes after stroke. Brain ischemia induces not only local inflammation but also systemic immune alterations. These systemic alterations exacerbate secondary complications such as infections and furthermore recurrent cardiovascular events. DAMPs, released from ischemic brain tissue, and binding to PRRs initiate a systemic inflammatory response to stroke.

Emerging studies suggest cfDNA as a very promising molecule within the large and heterogeneous group of DAMPs released after stroke. Cumulative evidence from clinical studies by now suggests that quantification of blood cfDNA concentrations might be valuable diagnostic biomarker for stroke with a predictive value for long-term outcome. The rapid release of large amounts of cfDNA after stroke presents an interesting and novel therapeutic candidate to ameliorate systemic inflammatory consequences after stroke. Various therapeutic approaches including cfDNA degradation, blocking its receptor interaction and inhibiting downstream signaling, are available and represent promising candidates for further translational validation.

In summary, cfDNA provides great potential as an acute fluid biomarker after stroke. Further investigations are needed to understand the full impact of cfDNA on local and systemic inflammation after stroke. Moreover, possible treatment approaches need confirmation from preclinical and clinical studies.
